# The difference of intestinal microbiota composition between Lantang and Landrace newborn piglets

**DOI:** 10.1186/s12917-023-03642-z

**Published:** 2023-09-27

**Authors:** Ling Li, Shuai Li, Junyi Luo, Ting Chen, Qianyun Xi, Yongliang Zhang, Jiajie Sun

**Affiliations:** College of Animal Science, Guangdong Provincial Key Laboratory of Animal Nutrition Control, National Engineering Research Center for Breeding Swine Industry, South China Agricultural University, Guangzhou, Guangdong 510642 China

**Keywords:** Breeds, Newborn piglets, Jejunal microbiota, High throughput sequencing

## Abstract

**Background:**

The early development of intestinal microbiota plays a fundamental role in host health and development. To investigate the difference in the intestinal microbial composition between Lantang and Landrace newborn piglets, we amplified and sequenced the V3-V4 region of 16 S rRNA gene in jejunal microbiota of Lantang and landrace newborn.

**Results:**

The findings revealed that the dominant phyla in the jejunum of Lantang piglets were Firmicutes, Actinobacteria and Bacteroidetes, while the dominant phyla of Landrace is Proteobacteria and Fusobacteria. Specifically, *Corynebacterium_1, Lactobacillus, Rothia, Granulicatella, Corynebacteriales_unclassified, Corynebacterium, Globicatella* and *Actinomycetales_unclassified* were found to be the dominant genera of Lantang group, while *Clostridium_sensu_stricto_1, Escherichia-Shigella, Actinobacillus* and *Bifidobacterium* were the dominant genera of Landrace. Based on the functional prediction of bacteria, we found that bacterial communities from Lantang samples had a significantly greater abundance pathways of fatty acid synthesis, protein synthesis, DNA replication, recombination, repair and material transport across membranes, while the carrier protein of pathogenic bacteria was more abundant in Landrace samples.

**Conclusions:**

Overall, there was a tremendous difference in the early intestinal flora composition between Landang and Landrace piglets, which was related to the breed characteristics and may be one of the reasons affecting the growth characteristics. However, more further extensive studies should be included to reveal the underlying relationship between early intestinal flora composition in different breeds and pig growth characteristics.

**Supplementary Information:**

The online version contains supplementary material available at 10.1186/s12917-023-03642-z.

## Background

Lantang (a Chinese indigenous lardtype breed) and Landrace (a typical lean breed) pigs show obvious differences in stress resistance, production, growth performances and meat quality characteristics [1]. In general, Lantang has high reproductive rate, strong adaptability and good meat quality [2]; Landrace is well-known for fast growing rate and lean meat percentage but low ability of resistance to disease [3]. The molecular mechanism underlying these phenotype differences has been well studied, and a growing body of evidence has also unraveled important roles of the gut microbiome in immune function [4], organ development [5], host metabolism [6] and colonization resistance to enteric pathogens [7].

Accumulating information about porcine gastrointestinal tract microorganisms has been acquired since the expansive application of “omics analysis” [8, 9]. Emerging studies deemed that the gut of newborns is short of microorganisms before birth, but will rapidly arise a distinct shift from a basically sterile state to an extremely dense microbial population [10, 11]. The colonization and succession of gut microbe in the early postpartum period facilitates immune maturation and have long-term impacts on the healthy growth and development of newborns [12]. In addition to many assorted environmental factors, the influence of breeds on the composition of early intestinal flora in piglets cannot be ignored [13].

The importance of the intestinal microbiota for early growth in pigs have been extensively studied, but fewer studies have investigated the possible association between breeds and early intestinal microbial composition of piglets [14].Therefore, the major aim of this study was to investigate the jejunal microflora of newborn between Lantang and Landrace, and to compare the characteristics of intestinal microflora involved in pig breeds.

## Result

### 16 S rRNA sequencing and annotation

**Fig. 1:**
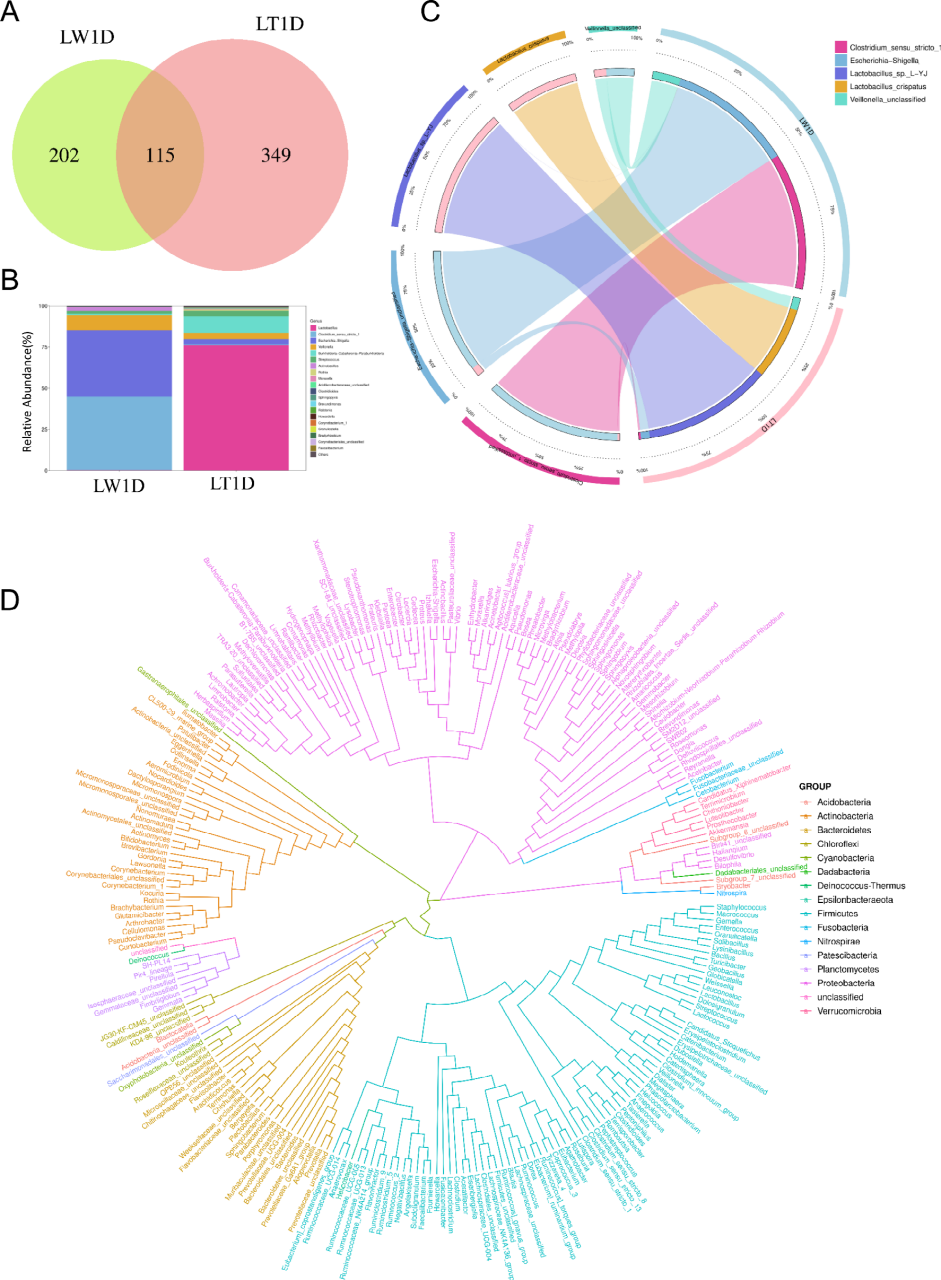
16 S rRNA gene sequencing and annotation analysis (**A**) Venn diagram. Venn diagram representing the shared and exclusive OTUs at the 97% similarity level between Jejunal microbiota in the two groups (**B**) Stacked bar chart. Bar plot shows the relative abundance of jejunal microbiota at the genus level in each group (**C**) Circos. The left half of circos is the top5 relative abundance species and their corresponding abundance information; the right half is the grouping information displays according to groups, and the wider the width, the higher the abundance (**D**) Phylogenetic tree. Different colors represent different phyla and different branches represent different genus levels. The closer the distance between the two species, the closer the evolutionary relationship between them Note: OTUs, operational taxonomic units; LW1D, Lantang piglets; LT1D, Landrace piglets

By high-throughput sequencing analysis, a total of 1,677,229 raw reads were generated from Landrace and Lantang libraries. Each library produced an average of approximately 83,861 joined tags (minimum = 80,032 and maximum = 87,907), and more than 90.47% valid reads from each sample were processed for further analysis (Table S1). Based on the obtained feature OTU, a total of 666 convincing candidates were identified (Table S2A); and only 115 candidates were shared across two groups, while 202 and 349 were identified uniquely in the Landrace and Lantang groups, respectively (Fig. 1A). All OTU candidates were annotated with the SILVA database to evaluate the microbial diversity in the jejunum of two breeds using the QIIME2 pipeline, which detected a total of 16 taxonomic phyla (Table S2B). In details, the dominant phyla in the jejunum of piglets were Firmicutes, Proteobacteria, Actinobacteria, Bacteroidetes and Fusobacteria, accounting for 70.25%, 29.15%, 0.53%, 0.03% and 0.02%, respectively. And the proportion of Firmicutes (84.58% vs. 55.92%), Actinobacteria (0.10% vs. 0.06%) and Bacteroidetes (0.05% vs. 0.01%) were greater in the jejunum of Lantang piglets than in Landrace, while Proteobacteria (14.31% vs. 43.98%) and Fusobacteria (0.01% vs. 0.02%) were lower in the jejunum of Lantang piglets (Figure S1). The number of 37 classes, 73 orders and 130 families were detected, of which four classes, six orders and seven families had a relative abundance greater than 1.0% (Table S2C, D, E). At the genus level, a total of 257 classifiable genera were identified (Table S2F), and seven genera had a relative abundance greater than 1.0%, including *Lactobacillus, Clostridium_sensu_stricto_1, Escherichia-Shigella, Veillonella, Burkholderia-Caballeronia-Paraburkholderia, Streptococcus and Actinobacillus* (Fig. 1B). Among them, *Lactobacillus, Burkholderia-Caballeronia-Paraburkholderia* and *Streptococcus* were found to be more abundant in the jejunum of Lantang piglets, while *Clostridium_sensu_stricto_1, Escherichia-Shigella, Veillonella*, and *Actinobacillus* are more abundant of Landrace. Among the 391 species detected (Table S2G), the most abundant species in the jejunum of Lantang was *Lactobacillus_sp_L-YJ*, and the most abundant species of Landrace was *Clostridium_sensu_stricto_1_unclassified* (Fig. 1C). In addition, the tree of species evolution was constructed according to the multi-sequence alignment results of feature sequences (Fig. 1D). We drew a conclusion that functional diversity of bacterial communities were represented mainly by a clade of Firmicutes, Proteobacteria, Actinobacteria and Bacteroidetes. In details, *Helicobacter* and *ruminococcaceae_ucg-005* from Firmicutes have a correlative evolutionary relationship, and *Gastranaerophilales_unclassified* is closely related to the evolution of Actinobacteria.

### The difference of intestinal microbiota composition between two breeds

**Fig. 2:**
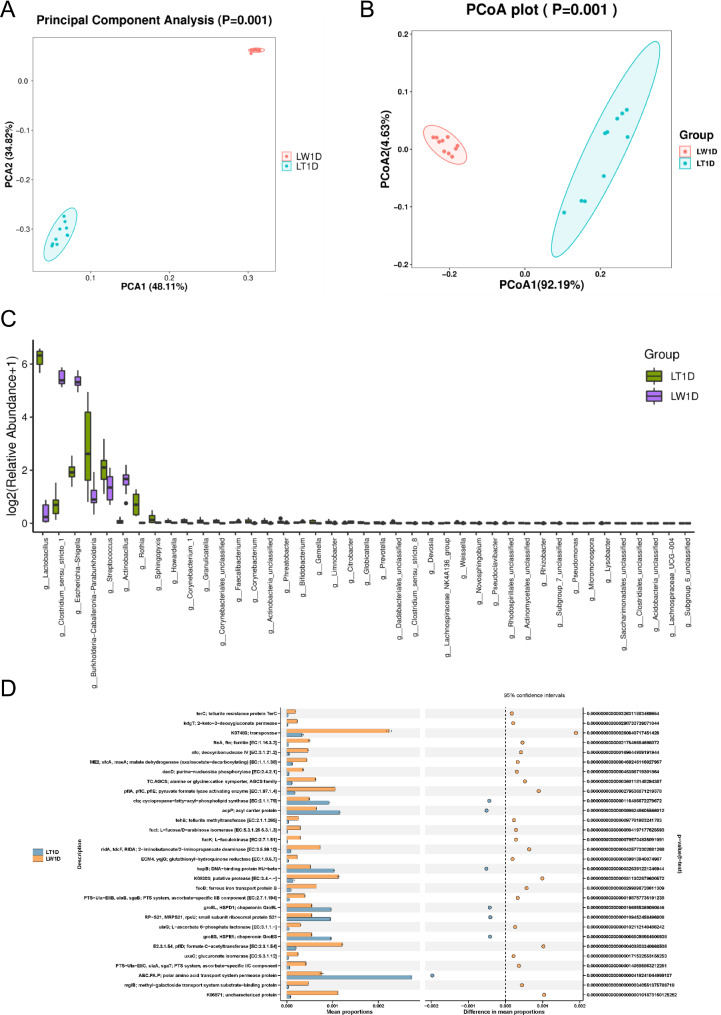
Microbial diversity in the jejunum of the two breeds (**A**) Principal Component Analysis (PCA). The same group is displayed as a circle graph with 95% confidence interval. The farther the distance between two samples, the greater the difference of microbial composition and structure between samples (**B**) Principal coordinate analysis (PCoA). The PCoA is based on the weighted UniFrac distance (**C**) Barplot difference analysis. The barplot shows microorganisms with significant differences at the genus level (P < 0.05 is defined as significantly different species) (**D**) Functional prediction STAMP difference analysis. The analysis results show the top 30 differential classification Clusters between the two group in KO function pathways (*P* < 0.05, 95% confidence interval) Note: LW1D, Lantang piglets; LT1D, Landrace piglets

The Observed species, Shannon, Simpson, Chao1, and Goods_coverage indices between two groups were calculated to compare the jejunum microbial alpha-diversity of the two breeds (Table 1). There was an increasing trend of Observed species (*P* = 0.08) and Chao1 (*P* = 0.09) diversity indices in the Lantang group compared with those of the Landrace group, and the Simpson diversity index exhibited a significant increase in the Landrace (*P* = 0.02). For the beta-diversity, the PCA (Fig. 2A), PCoA (Fig. 2B) and NMDS (Figure S2) analysis showed Lantang and Landrace samples could be separated absolutely into two groups, which reflected the differences in early jejunum microbial community composition between different breeds of piglets. And the cluster tree revealed the significant structural separation of jejunal microflora between the two breeds (Figure S3). Subsequently, LefSe algorithm was employed to identify statistically significant biomarkers among the two groups. A total of 117 differentially abundant taxonomic clades were found, with a Linear Discriminant Analysis (LDA) score higher than 3. The number of biomarkers at the phylum, class, order, family, genus and species levels were 4, 9, 15, 20, 26, and 43, respectively. Among them, 14 genera can be used as biomarkers for Lantang samples, including *lactobacillus*, *Burkholderia-Caballeronia-Paraburkholderia*, *Streptococcus*, *Novosphingobium*, *Subgroup_6_unclassified, Rothia*, *Weissella*, *Pseudoclavibacter*, *Subgroup_7_unclassified*, *Acidobacteria_unclassified*, *Rhizobacter*, *Actinomycetales_unclassified*, *Howardella*, *Pseudomonas*, while 12 genera can be used as biomarkers for Landrace samples, including *Actinobacillus*, *Lysobacter*, *Micromonospora*, *Lachnospiraceae_UCG-004*, *Lachnospiraceae_NK4A136_group*, *Rhodospirillales_unclassified*, *Faecalibacterium*, *Bifidobacterium*, *Clostridiales_unclassified*, *Citrobacter, Clostridium_sensu_stricto_1*, *Escherichia-Shigella* (Figure S4). According to the sample species abundance table, we found 5, 5, 11, 16, 12 and 28 significantly different microorganisms at the phylum, class, order, family, genus and species levels, respectively (Table S3). The difference analysis results displayed that the significantly differential microorganisms were mainly classified to the phyla of Firmicutes and Actinobacteri in Lantang group and the phyla of Proteobacteria and Firmicutes in Landrace group (Fig. 2C). Specifically, the dominant genera of Lantang group were *Corynebacterium_1, Lactobacillus, Rothia, Granulicatella, Corynebacteriales_unclassified, Corynebacterium, Globicatella* and *Actinomycetales_unclassified*, while the dominant genera of Landrace were *Clostridium_sensu_stricto_1, Escherichia-Shigella, Actinobacillus* and *Bifidobacterium.*

**Table 1:** Microbial diversity indices of the jejunal microbiome

Item	LW1D	LT1D	P value
Observed species	78.20 ± 8.64	113.30 ± 16.87	0.08
Shannon	2.86 ± 0.03	2.59 ± 0.22	0.25
Simpson	0.77 ± 0.01	0.65 ± 0.05	0.02
Chao1	80.38 ± 8.97	115.55 ± 17.42	0.09
Goods_coverage	0.99 ± 0.00	0.99 ± 0.00	1.00

Note: The values were calculated as the mean ± standard error of the mean. *P* < 0.05 indicated a significant difference between the two groups. LW1D, Lantang piglets; LT1D, Landrace piglets

In general, the relationship between different microbial groups plays a considerable role in revealing some biological significance. The reticulum network contained a node representing *Lactobacillus*. Its neighbors formed five mutually exclusive clusters: *Burkholderia-Caballeronia-Paraburkholderia* was positively correlated with *Lactobacillus*, and the other negatively correlated with *Lactobacillus*, consisting mainly of members of the *Escherichia-Shigella*, *Clostridium_sensu_stricto_1*, *Actinobacillus* and *Veillonella*. The genus of *Clostridium_sensu_stricto_1* was positively related to *Escherichia-Shigella*, *Actinobacillus* and *Veillonella*, and negatively related to *Burkholderia-Caballeronia-Paraburkholderia*. The genus of *Escherichia-Shigella* was observed to be positively correlated with *Actinobacillus* and *Veillonella*, and negatively correlated with *Burkholderia-Caballeronia-Paraburkholderia* (Figure S5).

### Molecular function of bacterial microbiota

PICRUSt2 software was employed to predict the functionality of the microorganism using the 16 S rRNA gene data. We found Abundant gene families were identified in all samples; of these, many of these genes play novel roles in glycolysis, fatty acid metabolism, amino acid metabolism, DNA replication and repair and pathogenicity (Table S4A and S4B). Prediction of these function revealed significant differences between the jejunal microbe of the two breeds. There were 4,231 and 2,718 differential functional features of the jejunal microbiome between the two breeds in the KO and COG database, respectively (Table S4C and S4D). We provided a visual representation of the top 30 secondary classification Clusters of KO pathways (Fig. 2D). The abundance of seven functional annotations in the Lantang group was higher than that in the Landrace group, including “cfa; cyclopropane-fatty-acyl-phospholipid synthase [EC:2.1.1.79]”, “acpP; acyl carrier protein”, “hupB; DNA-binding protein HU-beta”, “groEL, HSPD1; chaperonin GroEL”, “RP-S21, MRPS21, rpsU; small subunit ribosomal protein S21”, “groES, HSPE1; chaperonin GroES” and “ABC.PA.P; polar amino acid transport system permease protein”. The abundance of other 23 functional annotations was higher in the Landrace group, such as “terC; tellurite resistance protein TerC”, “kdgT; 2-keto-3-deoxygluconate permease”. For the COG pathways, only four functional annotations were higher in the Lantang group than in the Landrace group, which were the “Na+/H + antiporter NhaD or related arsenite permease”, “Co-chaperonin GroES (HSP10)”, “Uracil-DNA glycosylase” and “Cyclopropane fatty-acyl-phospholipid synthase and related methyltransferases” (Figure S6).

## Discussion

In this study, clustering analysis revealed that the Firmicutes, Actinobacteria and Bacteroidetes were the dominant phyla in Lantang piglets, while Proteobacteria and Fusobacteria were the dominant phyla in Landrace. In agreement with previous results, pig breed affects the proportion of Firmicutes and Bacteroidetes, which are higher in Chinese indigenous pig breeds than in foreign breeds [14]. Firmicutes, Actinobacteria and Bacteroidetes are the main energy-producing bacteria in the gut, decomposing various kinds of non-digestible polysaccharides and fermentating the resulting monosaccharides into SCFA, primarily acetate, propionate and butyrate, as well as other organic acids [15]. Additionally, the proportion of Firmicutes and Bacteroidetes have been attributed to energy metabolism and immune regulation [16–18]. However, there is increasing evidence that Proteobacteria is related to the dysbiosis and metabolic disorders [19, 20]. Fusobacteria has been supposed in the proinflammatory signature and disease of the body [21–23]. Hermann-Bank et al. [24] found that the number of Fusobacteria bacteria was doubled in piglets experiencing diarrhea.

Different pig breeds may exhibit their unique microbial diversity [25]. In this study, the PCA, PCoA and NMDS analysis indicated that Lantang and Landrace samples are mainly gathered into two different regions. This result corroborates the report of Yang et al. [14], who observed that the pig breeds affected the composition of intestinal microbiota, and the composition is different with different breeds, especially between Chinese indigenous breeds and foreign breeds. Genetic background is closely related to the intestinal microbial taxa and characteristics of the host [26].

Among the differential microbes of jejunum between the two breeds, *Corynebacterium_1, Lactobacillus, Rothia, Granulicatella, Corynebacteriales_unclassified, Corynebacterium, Globicatella* and *Actinomycetales_unclassified* was found to be more abundant in the jejunum of Lantang piglets, while *Clostridium_sensu_stricto_1, Escherichia-Shigella, Actinobacillus* and *Bifidobacterium* were more abundant of Landrace. *Corynebacterium_1*, *Rothia, Corynebacteriales_unclassified, Corynebacterium* and *Actinomycetales_unclassified* are all from phylum Actinobacteria. Binda et al. [20] suggested that Actinobacteria are absolute participants in preserving intestinal barrier homeostasis. Moreover, *Rothia* breaks down protein in feed and helps the digestion and absorption of nutrients in the intestine [27]. *Lactobacillus* is a major systematic group in the proximal gastrointestinal tract of several mammals [28, 29], which can metabolize carbohydrates to produce lactic acid, the end product [30, 31]. Previous studies reported that some species of *Lactobacillu*s could be transferred from the mother to the intestines of piglets via faeces or breast milk, so as to prevent and treat diarrhea [32]. Consistent with the findings of predecessors, the abundance of *Clostridium_sensu_stricto_1* and *Escherichia-Shigella* are high in suckling Landrace piglets, and they have been proved to be potential causative agents of diarrhea [33–35]. *Actinobacillus* equal as a primary pathogen in breeding sows and piglets [36]. The capacity to efficiently use mucus is a typical feature of *Bifidobacterium*, an intestinal genus that maintaining healthy intestines and preventing disease [37]. Furthermore, we found that *Burkholderia-Caballeronia-Paraburkholderia* was positively correlated with *Lactobacillus*, and the other negatively correlated with *Lactobacillus*, consisting mainly of members of the *Escherichia-Shigella*, *Clostridium_sensu_stricto_1*, *Actinobacillus* and *Veillonella*. *Burkholderia-Caballeronia-Paraburkholderia* shows a unique spectrum of antimicrobial activity and inhibited carbapenem-resistant Gram-negative bacterial pathogens [38]. The importance of *Veillonella* in mammal infections is uncertain, and they are generally considered to be of low virulence [39]. The results showed that the predominant species of Lantang group was genus of Lactobacillus, while the Landrace group were genera of *Clostridium_sensu_stricto_1 and Escherichia-Shigella*. We believe that the difference of the above microorganism might be one of the reasons why Lantang pigs are resistant to rough feeding and have strong adaptability, while Landrace pigs have weak physique and poor resistance to stress.

Furthermore, bacterial communities from Lantang samples had a significantly greater abundance pathways of fatty acid synthesis, protein synthesis, DNA replication, recombination, repair and material transport across membranes. Previous studies have revealed that the *cfa* gene encodes cyclopropane fatty acyl phospholipid synthase, which converts unsaturated fatty acids to their cyclopropane form [40]. The cyclopropane fatty acids in cell membrane is momentous for bacteria to adapt to the rapidly changing environment [41, 42]. In mammals, Acyl carrier protein (ACP) is a universal and highly conserved carrier of acyl intermediates during fatty acid synthesis [43]. DNA-binding protein HU-beta are involved in DNA replication, recombination and DNA repair [44]. Chaperone proteins, as an indispensable part of the protein-folding mechanism of bacterial cells, help maintain cellular homeostasis [45, 46], may also offset the harmful effects of mutations [47]. Tokuriki et al. [48] showed that chaperonin GroEL/GroES promotes the evolution of recombinant proteins. Small subunit ribosomal protein S21 also plays an important role in protein synthesis [49]. The ABC transporter system promotes the absorption of nutrients by bacteria [50], excretes substances that are not conducive to cell growth out of the cell, and also participates in signal transduction, protein secretion and antigen presentation in eukaryotes [51]. However, the abundance of “terC; tellurite resistance protein TerC” and “kdgT; 2-keto-3-deoxygluconate permease” functional annotation was higher in the Landrace group than Lantang group. Some studies have shown that the tellurite resistance gene operon is widely spread among pathogenic bacterial species [52]. And 2-keto-3-deoxygluconate permease is considered as a carrier protein for the fermentation of gluconate via 2-keto-3-deoxygluconate in many Clostridium bacteria [53]. Moreover, the relative abundance of “Na+/H + antiporter” and “Uracil-DNA glycosylase” functional annotation in the Lantang group was found to be higher than that in the Landrace group. The Na+/H + antiporters play a primary role in the maintenance of intracellular pH homeostasis and dynamic balance of cellular Na+, and in the regulation of cell volume [54]. The Uracil-DNA glycosylase for the removal of Uracil formed by incorrect input or mutation during DNA repair [55, 56]. The functional responses of jejunum microbiome of the above two breeds was significantly different. It is reasonable to assume that the difference of early microbial composition are one of the reasons for the different growth characteristics of Lantang and Landrace.

## Conclusion

Our findings revealed that there were tremendous differences in the early intestinal microflora composition between Lantang and Landrace newborn piglets due to breed characteristics. Furthermore, we found that these differences might be associated with growth characteristics of different breeds via the functional prediction of bacteria. However, more further extensive studies should be included to reveal the underlying relationship between early y intestinal flora composition in different breeds and pig growth characteristics.

## Materials and methods

### Sampling

All piglets were collected from a large-scale pig farm (Xinfeng County, Shaoguan City, Guangdong Province, China). After the sows farrowed, 10 healthy and purebred Lantang and Landrace male piglets (the piglets had no contact with the external environment and did not take breast milk) were slaughtered and digesta of jejunum were collected immediately; subsequently, a total of 10 intestinal contents in each breed were snap-frozen in liquid nitrogen and stored at -80 °C for further analysis. The experiment were approved and conducted under the supervision of the Animal Care Committee of South China Agricultural University.

### DNA extraction, PCR amplification and sequencing

Microbial genomic DNA was extracted from intestinal digesta using cetyltrimethylammonium bromide (CTAB) method, and the integrity of genomic DNA was assessed by 1% agarose gel electrophoresis and quantified with NanoDrop 8000 spectrophotometer (Thermo Fisher Scientific, Waltham, MA). According to the concentration, DNA was diluted to 1 ng/µL using sterile water. The intestinal bacterial 16 S rRNA gene was amplified by polymerase chain reaction (PCR) with specific primers (341 F: 5’-CCTACGGGNGGCWGCAG-3’; 805R: 5’-GACTACHVGGGTATCTAATCC-3’) targeting V3-V4 variable region [57]. After amplification, The products were purified by AMPure XT beads (Beckman Coulter, Danvers, MA) and quantified by Qubit (Invitrogen, Carlsbad, CA). The libraries were pooled subsequently at equal molar ratios, and sequenced on Illumina NovaSeq platform (LC Sciences, Hangzhou, China).

### Bioinformatics analysis

The quality control and chimeric sequence filtering were carried out to obtain high-quality clean reads by fqtrim (http://ccb.jhu.edu/software/fqtrim/index.shtml) and Vsearch softwares (https://github.com/torognes/vsearch). The clean tags of all samples were then clustered, and the tags with over 97% similarity were regarded as one operational taxonomic unit (OTU). According to SILVA (release 132) classifier, the feature sequences were annotated and normalized into different taxonomic levels [58]. Alpha diversity is applied in analyzing complexity of species diversity for a sample through 5 indices, including Chao1, Observed species, Goods coverage, Shannon, Simpson, and all these indices in our samples were calculated with QIIME2 [59]. For beta-diversity analysis, principal component analysis (PCA) was employed to reveal the simple rules under the intricate data background. Principal coordinate analysis (PCoA) and non-metric multi-dimensional scaling (NMDS) were performed to visualize the relationship between the samples. Unweighted pair group method with arithmetic means (UPGMA) is commonly considered as a method that effectively demonstrating good grouping and sample repeatability. Mann-Whitney U test was utilized for species difference test, and linear discriminant analysis effect size (LEfSe) was employed to find the biomarkers between different groups [60]. SparCC was performed to calculate the abundance of the top 30 microorganisms at the genus level, and the network diagram and correlation heat map were drawn based on the correlation and significance *P* value between the two dominant bacteria groups [61]. The PICRUSt2 was performed to predict the potential molecular functions of bacterial communities by obtaining the abundance of each predicted functional trait corresponding to normalized feature [62, 63]. These predictions are pre-calculated for genes in databases including KEGG Orthology (KO) and Clusters of Orthologous Groups (COG).

### Statistical analysis

The results of alpha-diversity and bacterial phenotype prediction were statistically analyzed by the T-test using SPSS software 17.0 (IBM Corp., Armonk, NY). All other statistical analyses were completed in R software (v3.4.4). Data were expressed as mean ± standard error of the mean (SEM), with *P* < 0.05 considered as statistical significance.

### Electronic supplementary material

Below is the link to the electronic supplementary material.

**Figure S1** Stacked bar chart. Bar plot shows the relative abundance of jejunal microbiota at the phylum level in each group. **Figure S2** Non-metric multi-dimensional scaling (NMDS). The NMDS analysis was based on the Bray–Curtis distance. Each point in the figure represents a sample, and the samples in the same group are represented by the same color. **Figure S3** On the left is the UPGMA cluster tree structure of each sample at the OTU level, and on the right is the relative abundance distribution map of each sample at the genus level. **Figure S4** Comparison of the classification of rumen microbiota between two groups by linear discriminant analysis effect size (LefSe) method. The LDA value distribution histogram (left) shows the species with significant differences in abundance in the two groups, and the length of the histogram represents the impact of different species. In the taxonomic cladogram (right), the circles radiating from the inside to the outside represent the classification level from phylum to species. **Figure S5** Sparcc network diagram and heat map. Different nodes in the network diagram represent different dominant genera. The connection between nodes indicates that there is correlation between the two genera. The thickness of the line indicates the strength of the correlation, and the size of the node indicates the number of other bacteria associated with the bacterium. **Figure S6** Functional prediction STAMP difference analysis. The analysis results show the top 30 differential classification Clusters between the two group in COG function pathways (*P* < 0.05, 95% confidence interval).

**Tables S1** Quality control and preprocessing of metagenomic datasets.

**Tables S2** (A) Sequence composition of each sample at each level based on the SILVA and NT-16S database. (B) The aligned percentages that annotated at Phylum level. (C) The aligned percentages that annotated at Class level. (D) The aligned percentages that annotated at Order level. (E) The aligned percentages that annotated at Family level. (F) The aligned percentages that annotated at Genus level. (G) The aligned percentages that annotated at Species level.

**Tables S3** (A) Differences between two groups identified at Phylum-taxa level. (B) Differences between two groups identified at Class-taxa level. (C) Differences between two groups identified at Order-taxa level. (D) Differences between two groups identified at Family-taxa level. (E) Differences between two groups identified at Genus-taxa level. (F) Differences between two groups identified at Species-taxa level.

**Tables S4** (A) Function prediction results in the COG database. (B) Function prediction results in the KO database. (C) Differential functional prediction analysis of each sample in in the COG databas. (D) Differential functional prediction analysis of each sample in in the KO database.

## Data Availability

The raw sequences were deposited into Sequence Read Archive (SRA) database with the BioProject accession number PRJNA876404 (https://www.ncbi.nlm.nih.gov/sra?LinkName=bioproject_sra_all&from_uid=876404).
