# Supplementary material for: The difference of intestinal microbiota composition between Lantang and Landrace newborn piglets
Source: BMC Vet Res. 2023 Sep 27;19:174. doi: 10.1186/s12917-023-03642-z (PMC10523759; doi:10.1186/s12917-023-03642-z)
Supplement: Supplementary file 1 — Figure S1 Stacked bar chart. Bar plot shows the relative abundance of jejunal microbiota at the phylum level in each group. Figure S2 Non-metric multi-dimensional scaling (NMDS). The NMDS analysis was based on the Bray–Curtis distance. Each point in the figure represents a sample, and the samples in the same group are represented by the same color. Figure S3 On the left is the UPGMA cluster tree structure of each sample at the OTU level, and on the right is the relative abundance distribution map of each sample at the genus level. Figure S4 Comparison of the classification of rumen microbiota between two groups by linear discriminant analysis effect size (LefSe) method. The LDA value distribution histogram (left) shows the species with significant differences in abundance in the two groups, and the length of the histogram represents the impact of different species. In the taxonomic cladogram (right), the circles radiating from the inside to the outside represent the classification level from phylum to species. Figure S5 Sparcc network diagram and heat map. Different nodes in the network diagram represent different dominant genera. The connection between nodes indicates that there is correlation between the two genera. The thickness of the line indicates the strength of the correlation, and the size of the node indicates the number of other bacteria associated with the bacterium. Figure S6 Functional prediction STAMP difference analysis. The analysis results show the top 30 differential classification Clusters between the two group in COG function pathways (P < 0.05, 95% confidence interval). [file 12917_2023_3642_MOESM1_ESM.docx]

Supplementary Figure


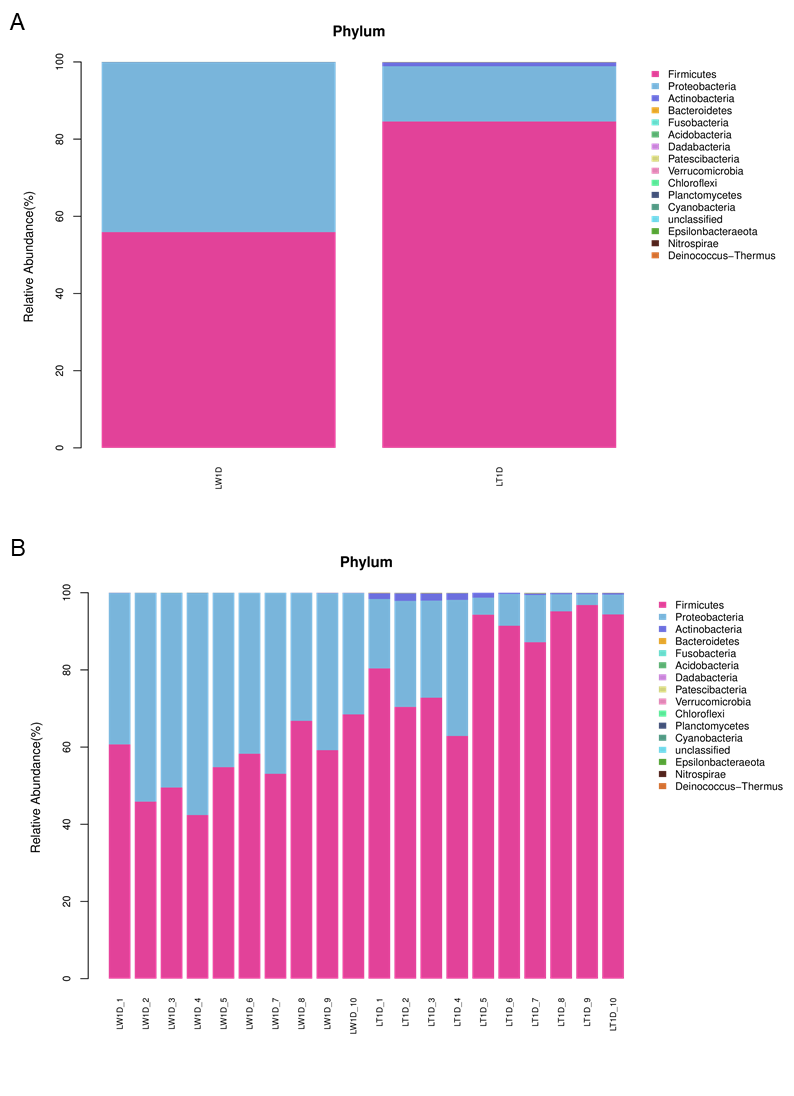


Figure S1 Stacked bar chart. Bar plot shows the relative abundance of jejunal microbiota at the phylum level in each group.


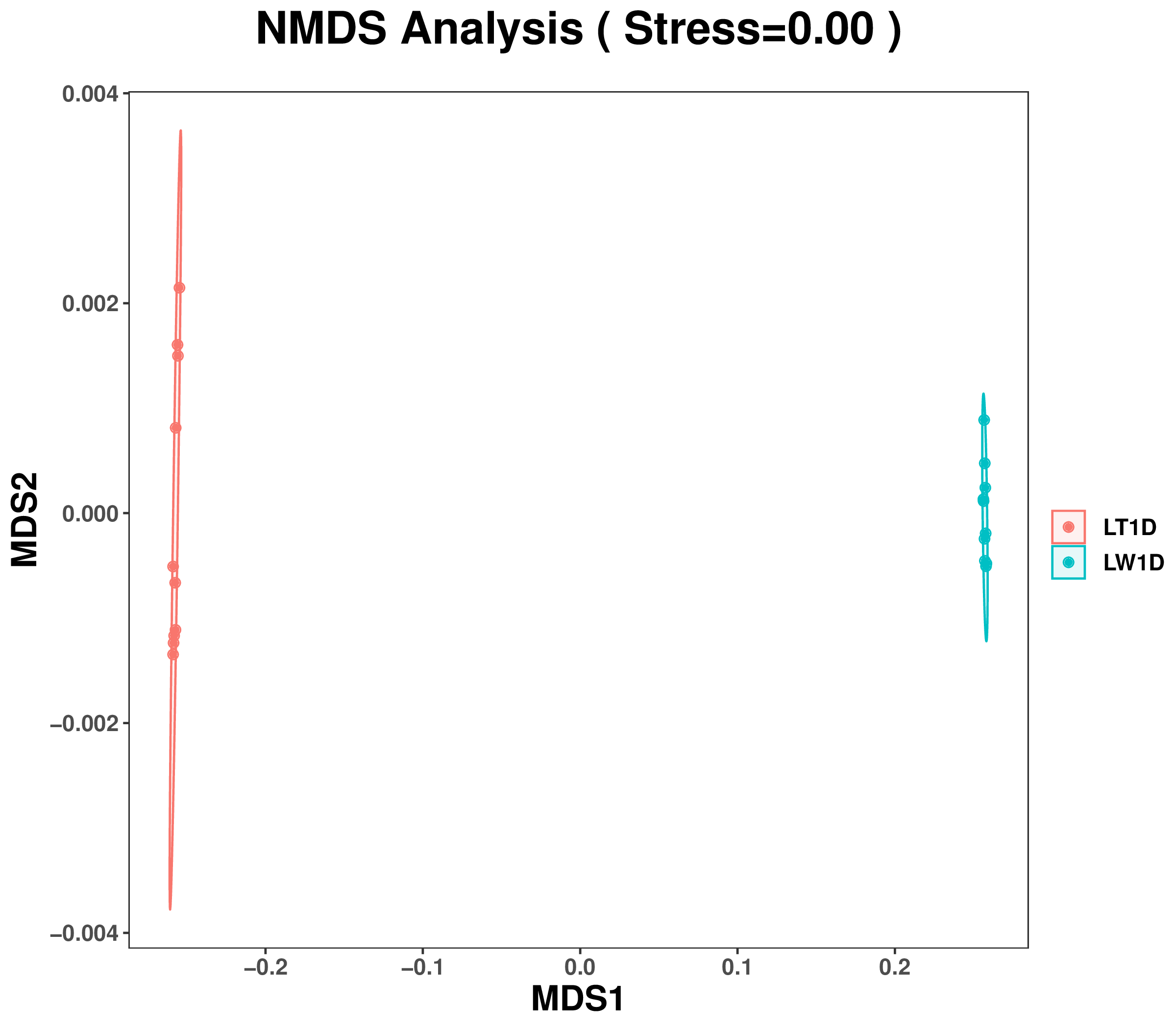


Figure S2 Non-metric multi-dimensional scaling (NMDS). The NMDS analysis was based on the Bray–Curtis distance. Each point in the figure represents a sample, and the samples in the same group are represented by the same color.


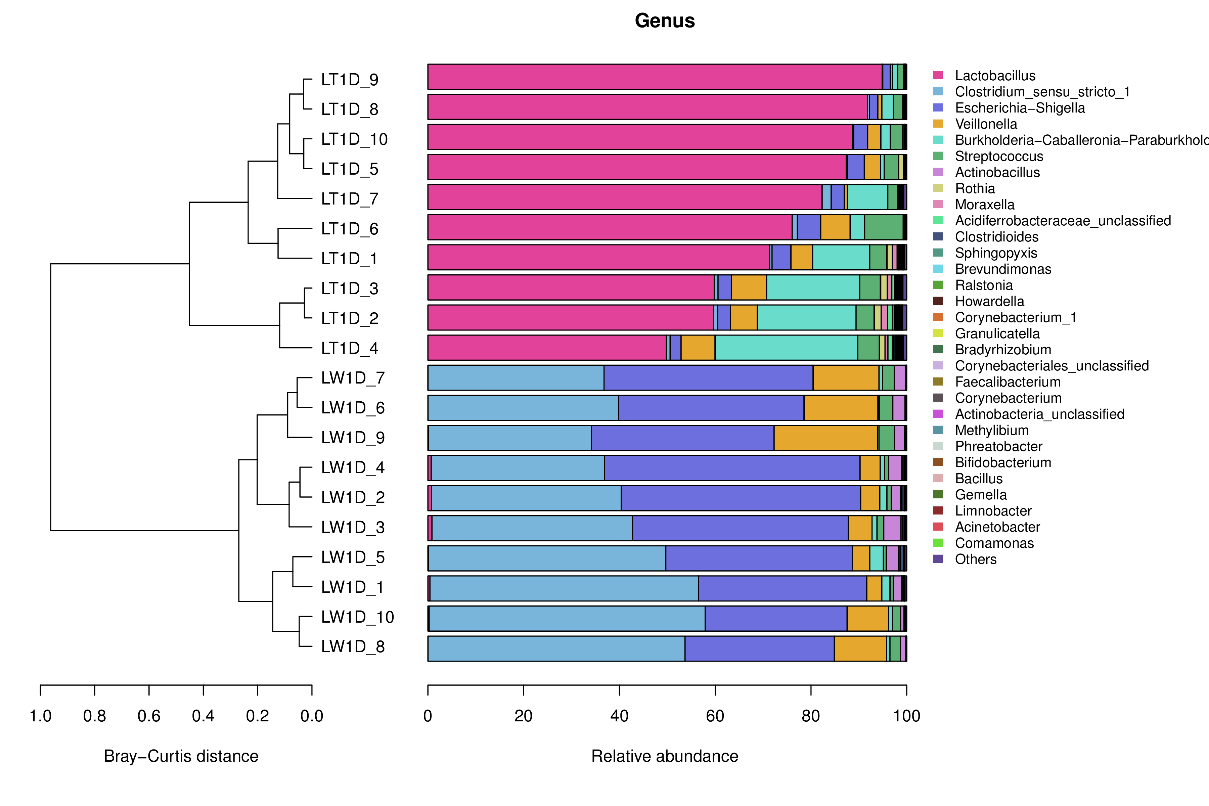


Figure S3 On the left is the UPGMA cluster tree structure of each sample at the OTU level, and on the right is the relative abundance distribution map of each sample at the genus level.


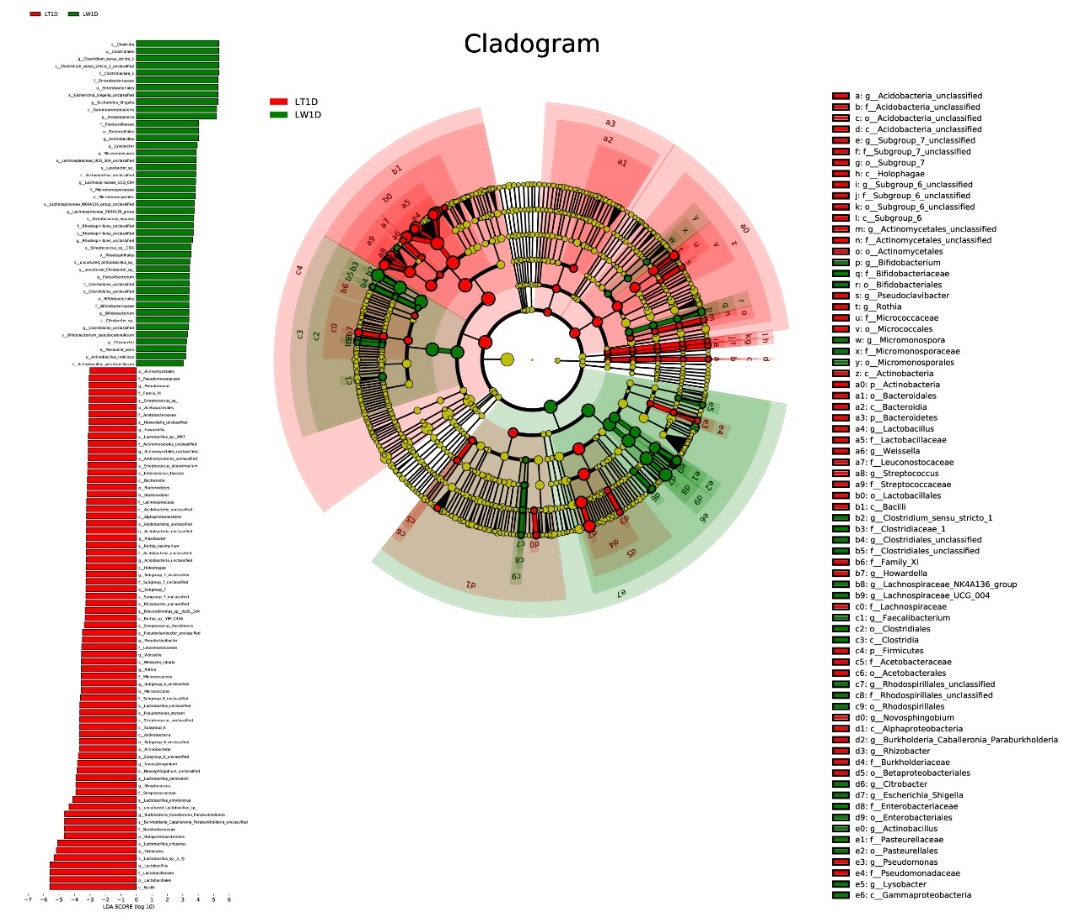


Figure S4 Comparison of the classification of rumen microbiota between two groups by linear discriminant analysis effect size (LefSe) method. The LDA value distribution histogram (left) shows the species with significant differences in abundance in the two groups, and the length of the histogram represents the impact of different species. In the taxonomic cladogram (right), the circles radiating from the inside to the outside represent the classification level from phylum to species.


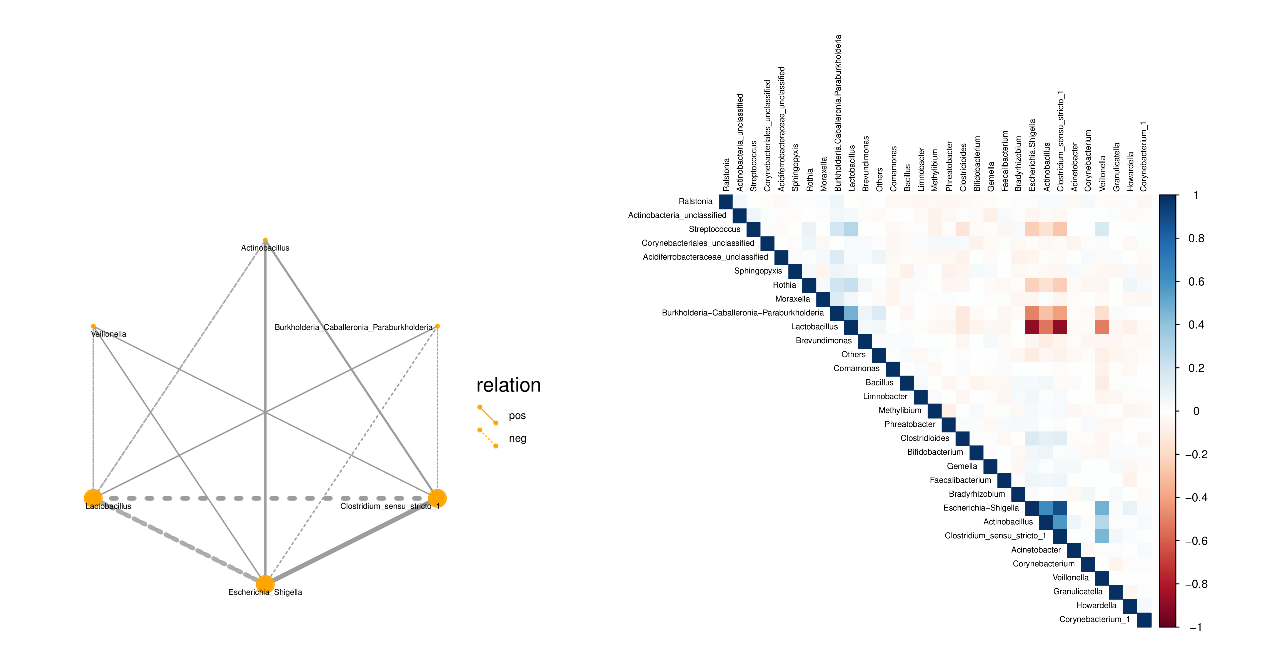


Figure S5 Sparcc network diagram and heat map. Different nodes in the network diagram represent different dominant genera. The connection between nodes indicates that there is correlation between the two genera. The thickness of the line indicates the strength of the correlation, and the size of the node indicates the number of other bacteria associated with the bacterium.


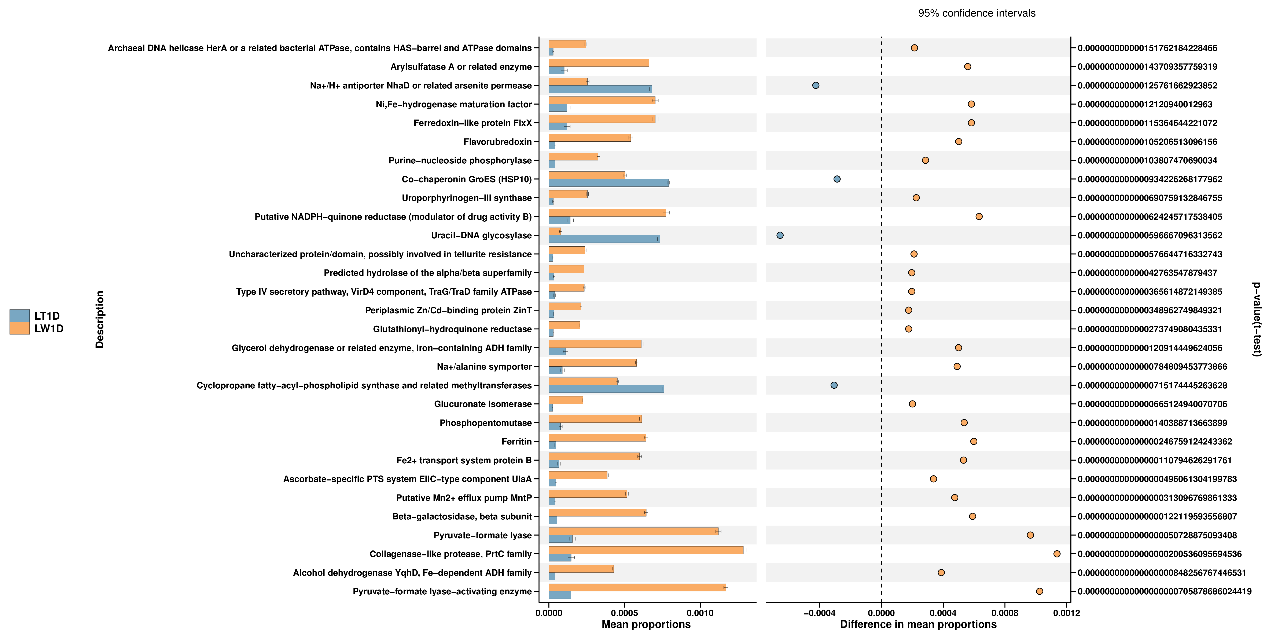
Figure S6 Functional prediction STAMP difference analysis. The analysis results show the top 30 differential classification Clusters between the two group in COG function pathways (*P* <0.05, 95% confidence interval).
